# Kidney transplants from elderly donors: what we have learned 20 years after the Crystal City consensus criteria meeting

**DOI:** 10.1007/s40620-024-01888-w

**Published:** 2024-03-06

**Authors:** Alberto Mella, Ruggero Calvetti, Antonella Barreca, Giovanni Congiu, Luigi Biancone

**Affiliations:** 1 Renal Transplant Center” A. Vercellone,” Nephrology, Dialysis, and Renal Transplant Division, “Città Della Salute e Della Scienza” Hospital, Department of Medical Sciences, University of Turin, Corso Bramante, 88, 10126 Turin, Italy; 2 Division of Pathology, “Città Della Salute e Della Scienza” Hospital, Department of Medical Sciences, University of Turin, Turin, Italy

**Keywords:** Expanded criteria donors, Kidney transplantation, Senescence, Discard rate, Machine perfusion

## Abstract

Based on the current projection of the general population and the combined increase in end-stage kidney disease with age, the number of elderly donors and recipients is increasing, raising crucial questions about how to minimize the discard rate of organs from elderly donors and improve graft and patient outcomes. In 2002, extended criteria donors were the focus of a meeting in Crystal City (VA, USA), with a goal of maximizing the use of organs from deceased donors. Since then, extended criteria donors have progressively contributed to a large number of transplanted grafts worldwide, posing specific issues for allocation systems, recipient management, and therapeutic approaches. This review analyzes what we have learned in the last 20 years about extended criteria donor utilization, the promising innovations in immunosuppressive management, and the molecular pathways involved in the aging process, which constitute potential targets for novel therapies.

**Graphical abstract:**

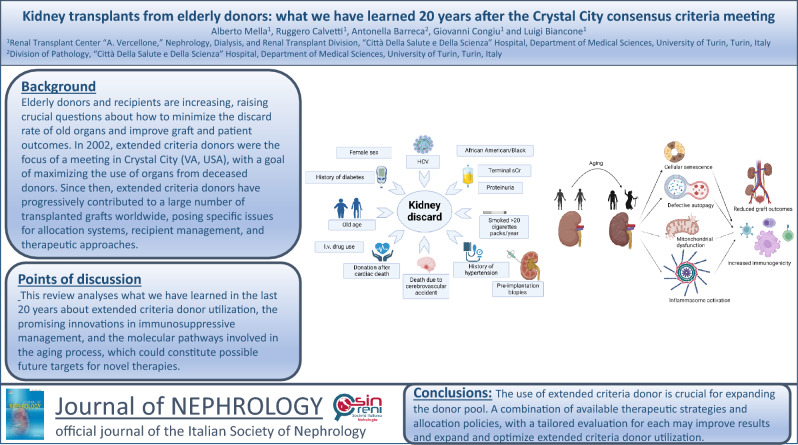

## Introduction

Kidney transplantation represents the best kidney replacement strategy as compared to all other dialysis options, and considering both clinical (morbidity/mortality) and socioeconomic profiles (quality of life, economic costs) [1]. Unfortunately, in the last decade, the number of patients with end-stage kidney disease has increased in parallel with life expectancy, widening the gap between potential transplant candidates and available organs [2].

Health systems worldwide struggle to increase the number of donors and use different approaches to deal with this problem. In particular, elderly donors now contribute to many transplanted grafts worldwide, posing specific issues for allocation systems, patient management, and therapeutic strategies [3, 4]. The exact definition and the consequent allocation policy of elderly donors are still debated. The Crystal City criteria provided the first consensus: all donors > 60 years old without comorbidities or > 50 years old with at least two conditions among high blood pressure, death by cerebrovascular accident, or serum creatinine levels > 1.5 mg/dL were classified as extended criteria donors [5]. Recently, policymakers in the United States adopted a different score based on 14 donor and transplant factors (the Kidney Donor Risk Index) to allocate grafts for single or dual kidney transplantation [6]. Despite all these strategies and increasing utilization (e.g., the number of donors ≥ 60 years old increased from 21% in 2000–2001 to 42% in 2016–2017 in the Eurotransplant senior program [7]), the balance between supply and demand is far from satisfactory, and many organs are still discarded [8].

This review discusses all the pros/cons of using extended criteria donor organs, focusing on optimal utilization, the potential innovations in immunosuppressive management, and the molecular pathways involved in the aging process and associated with graft dysfunction.

### Outcomes in recipients from extended criteria donors: good, bad, or (only) needing better allocation?

The increased use of marginal organs from elderly donors poses questions about their functional and clinical outcomes. Aubert et al. reported increased graft loss in patients who received organs from extended criteria donors (Hazard Ratio [HR] = 1.87 [1.50–2.32], p < 0.001 in multivariate analysis) compared to recipients of organs from standard criteria donors [9]. A meta-analysis by Querard et al. showed that both patient and death-censored graft survival were significantly better for recipients of standard criteria vs. extended criteria donor organs [10]. Additionally, Van Ittersum et al. highlighted higher death-censored graft failure and lower patient survival in recipients of organs from extended criteria donors vs. standard criteria/living donors [11].

Other authors documented similar rejection and death-censored graft survival rates at five years [12]. We recently revised our internal cohort of extended criteria donor recipients classified by decades of donor age, documenting similar patient (50–59 years old 87.8%; 60–69 years old 88.1%; 70–79 years 88%; > 80 years old 90.1%, *p* = 0.77) and graft (74.0%, 74.2%, 75.2%, 65.9%, *p* = 0.62) survival at five years. Considering that organs were allocated to single- or dual-kidney transplantation after a multistep evaluation including clinical and histological criteria, we investigated differences in the transplant outcomes and discard rate between groups, noting a better survival rate for dual-kidney transplantation from extended criteria donors > 80 years old (*p* = 0.04) and an increased number of kidneys discarded in this group (48.2%, Odds ratio [OR] 5.1 vs. 15.4%, 17.7% and 20.1% in other decades) [13]. On the basis of this experience, it would appear that appropriate selection provides comparable long-term outcomes in recipients of extended criteria donor organs, even considering the adoption of dual-kidney transplantation from very old donors (i.e., > 80 years old).

Although the long-term efficacy may be questioned, receiving a kidney from an extended criteria donor demonstrates a benefit in survival rate compared to being kept on the waiting list [14, 15]. This difference is particularly notable for recipients > 60 years old, for whom the survival-positive balance was approximately 15% (83.6% vs. 67.4%) [16], consistent with data from the United States [17].

More recently, Perez-Saez et al. confirmed this survival benefit (adjusted risk of death after transplantation, 0.44 [Confidence Interval (CI) 0.61–0.32; *p* < 0.001]) also in recipients of kidneys from deceased donors aged ≥ 75 years old, with acceptable death-censored graft survival (68.3% at ten years) [18]. It is worth mentioning that extended criteria donor recipients experienced death with functioning graft as the first cause of allograft loss [13, 19], stressing the influence of recipient factors in the outcomes and the risk of non-extended criteria donor utilization in lengthening the time on the waiting list for patients not highly suited for these organs (e.g., retransplant) [20].

These results highlight the need for flexible allocation policies that, taking into account the longevity of the transplanted kidneys, primarily offer organs from nonstandard criteria donors, extended criteria donors, or high Kidney Donor Risk Index donors to eligible elderly recipients and balance the pros and cons in an aging population, the increasing number of subjects with comorbid conditions, the expected benefit of extended criteria donors compared to standard criteria/living donors, and the risk of patient persistence on the waiting list. A proper allocation system could maximize the pros and reduce the cons (see Table 1), even by considering specific allocation programs according to geographical area (e.g., opt-in/opt-out policy for kidneys donated after brain death/circulatory death) and highlighting some comorbidities or parameters that may be helpful for appropriate post-transplant monitoring [21]. For example, in our experience, recipients with type 2 diabetes mellitus may have a worse outcome with extended criteria donor organs compared to non-type 2 diabetes mellitus patients, especially when receiving grafts from > 70-year-old donors [22], and recipients with pre-existing hypotension (mean blood pressure < 80 mmHg) had worse death-censored graft survival when receiving a transplant from donors > 50 years old [22]. Additionally, post-transplant proteinuria represents a crucial determinant of adverse outcomes in recipients of organs from donors > 50 years old [23].

**Table 1:** Advantages and disadvantages of extended criteria donor utilization

Extended criteria donor use-associated risks	Extended criteria donor use-associated benefits
Potential increased graft loss vs. standard/living donors [9–11]	Expansion of donor pool [19, 20]
Controversial survival advantage in recipients of extended criteria donor retransplantation [20, 24]	Better utilization for standard/living donors for high-risk patients [19, 20]
Reduced death-censored graft survival when receiving a transplant from a donor > 50 years old in recipients with pre-existent hypotension (mean blood pressure < 80 mmHg) [22]	Similar outcomes when properly allocated by age [12] also considering dual kidney transplantation for donors > 80 years [13]
Potential risk of increased rehospitalization [25]	Significant survival benefit in patients receiving organs from extended criteria donors vs. remaining on the waiting list [14, 15] (especially for recipients > 60 years old) [16, 17], even from deceased donors aged ≥ 75 years old [18]
Controversial utilization in recipients < 50 years [26]	
Differences in time to equal risk of death compared to standard/living donors [15]	

For many conditions in the transplantation setting, extended criteria donor use is a balance between risks and benefits that starts from a proper allocation to guarantee the most appropriate match between patient and kidney

### Proper evaluation of extended criteria donors: discard rate and the role of pre-implantation biopsy

Any improvement in the use of extended criteria donor organs is centered on correct evaluation, trying to correctly answer the question of how a kidney could be used and when it should be discarded.

**Fig. 1:**
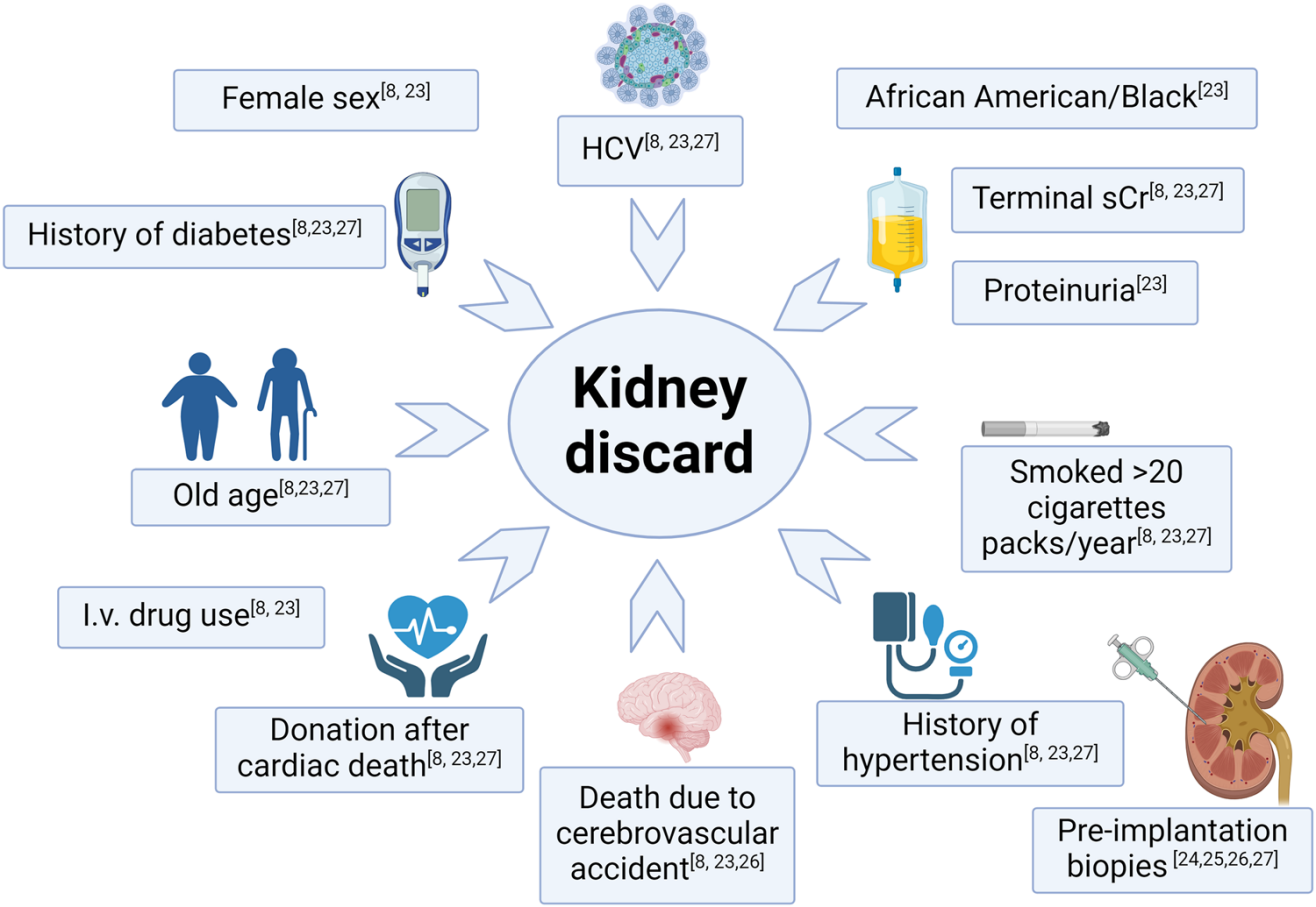
Leading determinants of increased discard rate in deceased donors. Created with BioRender.com

As mentioned above, kidney discard is a significant worldwide problem: as summarized in Fig. 1, literature data reflect a prevailing discard rate in older donors who are Black, female, diabetic, or hypertensive and those with undesirable social behavior and higher terminal creatinine level before donation [8, 27–31].

Mohan et al. [27] also raise the issue that pre-implantation biopsy findings are still the most commonly reported reason for discard, as documented by other reports [28, 29]. To date, pretransplant histological evaluations have been widely proposed and performed [32–34]. Nevertheless, concerns remain about the methods adopted to obtain and process material, the scoring system, and even the need for the biopsy itself.

In the methodology assessment, wedge biopsies, despite having a theoretically increased risk of complications compared with a needle biopsy, tend to overestimate the glomerulosclerosis rate (higher in the subcapsular cortex with correlation to discard if ≥ 20 [35]) with limited evaluation of arteries [36]. Needle biopsies or punch biopsies are more commonly used, with differences in the risk of bleeding or the sample size/accuracy [33].

A crucial point that is often not considered is the degree of experience of the nephropathologist: some studies have documented that on-call pathologists with limited experience with kidney biopsies assigned higher scores for chronic changes, leading to an increased number of discarded kidneys [36, 37].

From the initial grading system, which considered only glomerulosclerosis, and subsequent data showing the need for more extensive evaluation, the most widely adopted approaches now combine glomerular sclerosis, arteriosclerosis, hyaline arteriolosclerosis, and interstitial fibrosis in a grading system such as Karpinski’s score [38]. Each parameter receives a semiquantitative score of 0–3, and only kidneys with a cumulative score of ≥ 7 are discarded [38, 39].

The adoption of histological scoring systems combined with clinical and surgical parameters allows transplant teams to safely allocate extended criteria donor kidneys in single or dual transplantation with favorable outcomes [32, 33]. However, the logistical setting of the procurement area or the lack of centralized pathological evaluation may limit this approach, expanding the risk of prolonged cold ischemia time, which is the primary determinant, along with de novo donor-specific antibodies, of allograft function in extended criteria donor recipients [40].

This issue emphasized the desire to understand if there are specific clinical parameters that alone would best evaluate extended criteria donors and mitigate their discard rate [35, 41, 42]. The identification of reliable clinical parameters is a priority, especially since recent studies have already reported positive results with donors of very advanced age (> 70 or > 75 years) [18, 19].

The Eurotransplant consensus found that kidneys from 65- to 74-year-old donors can also be allocated to 55- to 64-year-old recipients without pre-implantation biopsies. This allocation was particularly recommended if kidneys were derived from donors without hypertension, increased creatinine, cerebrovascular death, or other reasons for definition as a marginal donor, such as diabetes or cancer [7].

The Kidney Donor Risk Index (adopted before the Kidney Donor Profile Index) considers donor age, height, weight, ethnicity, history of hypertension or diabetes, cause of death, serum creatinine, history of hepatitis C, and donation after cardio-circulatory death as clinical parameters for donor evaluation before allocation, stressing their importance in post-transplant graft survival [6]. However, some studies suggest that the application of the Kidney Donor Risk Index and Kidney Donor Profile Index may have resulted in an overestimation of high-risk organs, leading to excessive discard, and pose some questions about a decision based solely on clinical criteria (especially for some variables such as serum creatinine at the time of donation) [43].

In our opinion, biopsy findings in a favorable setting, such as that of our center, (i.e., limited kidney processing time, expert pathologists available 24/7) maintain their role in assessing kidney graft prospects and baseline pre-transplant damage, implementing clinical information without constituting the only parameter for discarding kidneys, as suggested by other authors [19]. These data could also be further implemented by artificial intelligence/computer-assisted evaluation of histological sections and acute kidney injury biomarkers such as neutrophil gelatinase-associated lipocalin to improve their significance and reliability [44–47]. Defining a clear profile to reject/retain organs is challenging, but a feasible approach with in-depth analysis of available histological/clinical profiles may also limit the discard rate for donors aged > 80 years, possibly considering dual kidney transplant in cases with suboptimal kidney function/comorbidities (i.e., hypertension/diabetes).

### Strategies to improve outcomes in recipients of extended criteria donor organs: machine perfusion

Organs from extended criteria donors are more prone to ischemia–reperfusion injury, with a consequent increased risk of delayed graft function [48].

In this context, adopting machine perfusion techniques may be an exciting strategy for reducing damage caused by ischemia–reperfusion injury, analyzing potential biomarkers of acute injury during perfusion, and applying reconditioning protocols [49]. Machine perfusion may also integrate clinical/histological information, as is already the case, with promising results in mitigating extended criteria donor discard [19].

Presently, two approaches are available: normothermic and hypothermic perfusion.

Normothermic machine perfusion has attracted increasing interest in recent years because it offers the advantage of a normal biochemical situation for evaluating graft function during perfusion and administering drugs to recondition organs. Some reports suggest that normothermic machine perfusion is beneficial in reducing ischemia–reperfusion injury and delayed graft function [50, 51]. Regarding the disadvantages of its utilization, current normothermic machine perfusion protocols require constant monitoring, continuous oxygenation with blood or other O_2_ carriers, and administration of nutritional supplements, with the additional risk of graft discard in case of pulse failure.

The second option, hypothermic machine perfusion, is, to date, simple, cost-effective, and applicable on a large scale without risk of graft loss in case of pump failure [52]. A preclinical transplantation study in pigs showed that hypothermic machine perfusion improved survival, chronic inflammation, epithelial to mesenchymal transition, and fibrosis markers [53]. Many real-life reports, including a recent meta-analysis, confirm the positive results of this technique compared to standard perfusion, which include reduced delayed graft function or primary nonfunction occurrence and increased allograft survival [54]. In Tingle et al., hypothermic machine perfusion resulted in better outcomes than standard cold storage in post brain and cardiac death donation; interestingly, in donation after cardiac death per se associated with an increased risk of delayed graft function, fewer perfusions were required to prevent a delayed graft function episode [55].

### Strategies to improve outcomes in recipients of extended criteria donor organs: tailored immunosuppression

The goal of immunosuppression in recipients of extended criteria donor grafts is to optimize the outcome and reduce the risk of clinical complications (i.e., infections, cancer), considering their higher immunogenicity (as discussed below), which exposes them to acute rejection episodes. This is even more critical in older recipients, in whom acute rejection incidence is generally lower but can lead to graft loss more frequently [56].

With regard to induction protocols, recent observations and a Cochrane meta-analysis reported the role of rabbit anti-thymocyte globulin vs. IL-2 receptor antagonists (e.g., basiliximab, daclizumab) in preventing acute rejection [57]. This effect is also demonstrated in elderly donors, in whom rabbit anti-thymocyte globulin has shown a lower risk of acute rejection than IL-2 receptor antagonists without an increased risk of death in older recipients and high-risk kidneys [58]. Gill et al. found that the adjusted odds of acute rejection at one year and mortality in kidney transplant recipients ≥ 60 years old were significantly higher among basiliximab recipients than rabbit anti-thymocyte globulin recipients [59]. Recently, Ahn et al. confirmed that rabbit anti-thymocyte globulin was associated with a decreased risk of acute rejection compared to basiliximab in both younger and older recipients; in younger recipients, rabbit anti-thymocyte globulin was also associated with a shorter time-to-discharge and reduced mortality risk compared with basiliximab [60].

Regarding maintenance therapy, kidney-transplanted patients, even elderly ones, most commonly receive triple therapy composed of tacrolimus/cyclosporine A, an antimetabolite (usually mycophenolate mofetil), and steroids [61, 62]. Interestingly, based on OPTN/UNOS data, Lentine et al. noted low adoption of depletion agents (rabbit anti-thymocyte globulin /alemtuzumab) in different combinations vs. basiliximab and more pronounced use of cyclosporine A-based immunosuppression induction in the older group (recipients > 65 years), with increased death-censored graft survival in patients without antimetabolite- or cyclosporine A-based regimens vs. standard treatment (induction with rabbit anti-thymocyte globulin /alemtuzumab followed by triple therapy) [61]. In a large European cohort of patients ≥ 60 years old, Echterdiek et al. showed similar 3-year death-censored graft loss and patient mortality between tacrolimus- and cyclosporine A-treated patients (in both cases with antimetabolite ± steroids) with a similar risk of hospitalization for global and bacterial infection but a lower incidence of rejection in tacrolimus-treated patients. Only BK virus infection and post-transplant diabetes were more prevalent in the tacrolimus group [62].

However, many studies suggest minimizing calcineurin inhibitor use based on the supposed increased susceptibility of older organs to higher levels of these drugs [63]. On the other hand, low/very low doses of calcineurin inhibitors or avoiding their use altogether may expose these increased immunogenic organs to a non-tolerogenic milieu, with a higher risk of acute rejection and donor-specific antibody production, clearly documented as a prevalent risk factor for graft failure in recipients of extended criteria donor organs [40, 64].

Mammalian target of rapamycin inhibitors (mTORi) have been proposed primarily in this context to avoid calcineurin inhibitor nephrotoxicity. However, large randomized clinical trials are not available [65], and despite some studies reporting positive results [58], mTORi utilization in patients receiving extended criteria donor organs remains a matter of debate [66], considering the documented risk of acute rejection in patients receiving tacrolimus + everolimus vs. the standard of care (tacrolimus + mycophenolate mofetil) [67].

Belatacept, a blocker of the costimulatory CD28/CD80 pathway [68], demonstrated a positive effect in increasing kidney function after conversion from calcineurin inhibitors in extended criteria donor organ recipients in the BENEFIT-EXT trial, with a 15 ml/min/1.73 m^2^ gain in belatacept-treated groups at seven years [69].

At the same time, some reports suggest positive results in extended criteria donor patients who switched from calcineurin inhibitors to Belatacept within the first six months post-transplant [70].

Some authors also noted an increased rejection rate in patients who switched from calcineurin inhibitors to Belatacept [71], but as also documented by our group, hybrid approaches with calcineurin inhibitor minimization (rather than avoidance) may reduce acute rejection risk, maintaining the positive belatacept effect on estimated glomerular filtration rate [72].

As shown in Table 2, each type of therapy may have a rationale and documented pros/cons based on literature data. In our opinion, a tailored approach should be applied for every patient based on their specific pre-transplant characteristics (i.e., age, immunological profile, years of dialysis) and the available information on the extended criteria donor kidneys. Considering the need to lower the discard rate and maximize post-transplant outcomes, excessive dependence on HLA matching to reduce the risk of de novo donor-specific antibodies may be adequately replaced by rabbit anti-thymocyte globulin induction and the adoption of triple therapy in standard patients, considering mTORi or the reduction of immunosuppressive load in a specific context according to the recipient’s history (e.g., history of cancer, cardiovascular/infectious risk), and belatacept + low tacrolimus in patients with delayed graft function or insufficient graft recovery.

**Table 2:** Pros and cons based on literature data among available and future strategies for immunosuppressive management in recipients of extended criteria donors

Rationale	Therapy/Regimens	Pros	Cons
Reduction of the increased rejection risk of extended criteria donors Steroid-sparing regimens	*Induction with rATG*	Lower risk of acute rejection vs. IL-2 receptor antagonists without an increased risk of death in older recipients and high-risk kidneys [58–60]	Higher death with functioning graft due to cumulative rATG dosage > 6 mg/kg [73]
	*TAC based-therapies*	Better death-censored graft survival for rATG + TAC/MMF/steroids graft survival vs. patients without antimetabolite or CyA-based regimens [61] Similar 3-year death-censored graft loss and bacterial infection but low-rejection vs. CyA-treated patients [62]	Increased risk of BK virus infection and post-transplant diabetes [62]
Minimization of CNI nephrotoxicity	*CNI-delayed introduction*	No increased risk of acute rejection adopting delayed reduced CyA doses/MMF/steroids + , IL-2 receptor antagonists induction [74]	No advantage in preserving renal function or reducing delayed graft function in older kidney transplant patients [75]
	*mTORi based-therapies*	Similar graft survival, acute rejection rates, and significantly better renal function at six months (no differences after that) [63]	Higher delayed graft function and acute rejection episodes with lower death censored graft survival and renal function in sirolimus vs. CyA [66] Increased incidence of acute rejection, graft loss, death, and treatment discontinuation with lower renal function in the rATG + everolimus group [67]
	*Belatacept-based therapy*	Similar patient/graft survival, better renal function, and improved cardiovascular/metabolic risk profile vs.CyA [76, 77] Renal function improvement in extended criteria donor patients who switched from CNI to Belatacept within the first six months [70]	Increased incidence of post-transplant lymphoproliferative disorders in patients negative for Epstein-Barr virus [76, 77] Increased rejection rate in patients switched from CNI to Belatacept [71]
Minimization of CNI nephrotoxicity Reduction/abrogation of immunosuppressive therapy after tolerance Organ reconditioning	*Cellular therapies (Treg, CAR-T, MSC, or MSC-EV)*	Anecdotal reports of good patient and graft survival and, apparently, low infection/rejection rates with Treg [78, 79] Potential favorable and protolerogenic microenvironment after MSC infusion [80, 81] Reduced allograft rejection with CAR-T in mouse models [82] with significant response in diminishing de-novo DSAs and frequencies of de-novo DSA-secreting B cells [83]	Significant variability between donor/patient characteristics with limited sample size [78, 79] Potential risk of malignant transformation for MSCs [80, 81] No tolerance achievement with Tregs alone [84, 85] No effect of CAR-T in sensitized mice (limited efficacy on memory alloresponse?) [83]

*rATG* rabbit anti-thymocyte globulin; *CyA* Cyclosporine A; *TAC* tacrolimus; *CNI* calcineurin inhibitor; *MMF/MPA* mycophenolate mofetil/mycophenolic acid; *mTORi* mammalian target of rapamycin inhibitor; *Tregs* T regulatory cells, *CAR-T* chimeric antigen receptor T cells; *DSAs* donor-specific antibodies; *BM-MSC* bone marrow-derived mesenchymal stromal cell; *MSC-EV* mesenchymal stromal cell-derived extracellular vesicle

### Future perspectives: cellular therapies

With regard to future approaches, cellular therapies have received progressively more and more attention in the last ten years and may theoretically allow, in older donor settings, achievement of immunological tolerance (thereby abolishing the need for nephrotoxic immunosuppressive drugs) and/or reconditioning/recellularizing donors with suboptimal kidney function.

Several approaches have thus far not demonstrated a significant benefit. In the TAIC-I trial, donor-derived transplant acceptance-inducing cells, a type of immunoregulatory macrophage, were administered as an adjunct immune conditioning therapy; eight out of ten kidney transplant recipients in whom immunosuppression was tapered tolerated steroid discontinuation, with an additional reduction of sirolimus/tacrolimus monotherapy in some cases. Nevertheless, the trial could not provide evidence that postoperative transplant acceptance-inducing cell administration has a documented ability to dampen allogeneic rejection [86].

Promising examples are derived from some T regulatory cell (Treg) trials, showing good patient and graft survival and, apparently, low infection/rejection rates [78, 79]. However, adopted protocols and donor/patient characteristics vary greatly among studies. The sample size was obviously scarce, and some intrinsic specificities of these cells (difficult isolation, effective homing in target sites) may limit their utilization. Notably, Treg infusion alone was insufficient to achieve tolerance, and combined immunosuppressive regimens are still under investigation [84, 85].

One interesting way to adapt the immune system involves synthetic chimeric antigen receptor cells that could be targeted toward donor HLA mismatches to redirect Treg specificity. In mouse allograft models, donor-specific chimeric antigen receptor Tregs effectively reduced allograft rejection [82]. More recently, they showed a striking ability to diminish de novo donor-specific antibodies and frequencies of de novo donor-specific antibody-secreting B cells but had no effect in sensitized mice, suggesting limited efficacy on memory alloresponse [83].

Bone marrow-derived mesenchymal stromal cells have also emerged in this field for their regenerative and tolerance-inducing potential. Many transplantation models and seminal trials, with a wide range of adopted cells and combined immunosuppression, have shown mixed results with the potential induction of a favorable and protolerogenic microenvironment after mesenchymal stromal cell infusion [80, 81]. Although their exact mechanism of action is not clearly understood, the greatest part of their protective and regenerative role could be mediated by indirect modulation of immune system components (e.g., macrophages, monocytes), as documented by similar results obtained through mesenchymal stromal cell-derived extracellular vesicles or conditioned medium infusion [87]. Although it seems that mesenchymal stromal cells have a very short lifespan in recipients with a lower risk of malignant transformation, using mesenchymal stromal cell-extracellular vesicles could overcome this problem. Nevertheless, available protocols must be refined to fulfill the quality and quantity requirements for practical application [88].

Complete chimerism with cessation of immunosuppressive drugs and tolerance of transplanted organs has been tested and obtained, but safety issues that presented after required immune system reconditioning limit this strategy [89].

### Aging at the cellular level: senescence in the kidney

Despite being partially questioned, donor age remains a critical factor in the long-term outcome of kidney transplantations, and extended criteria donor organs also carry an increased risk of acute rejection [56].

**Fig. 2:**
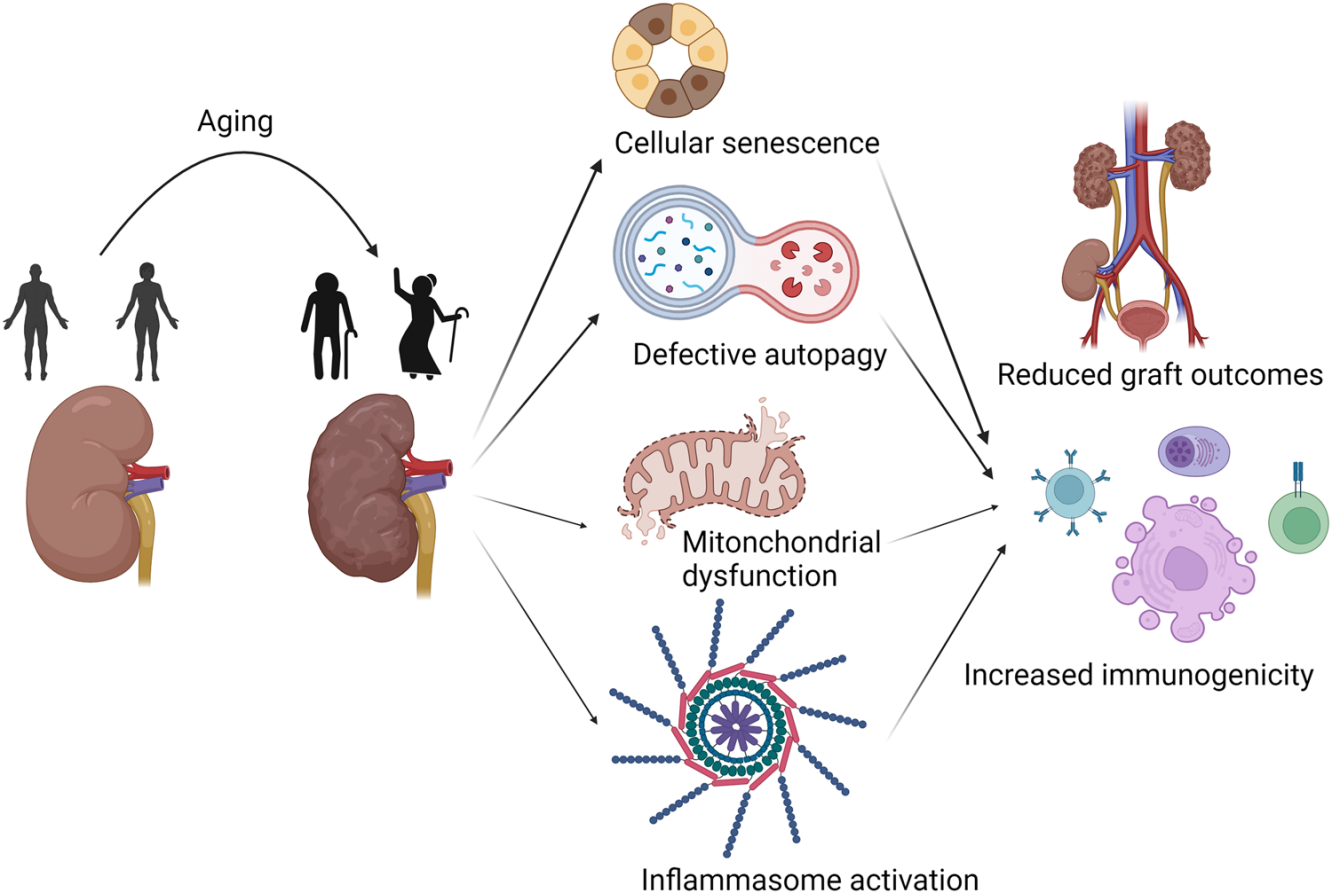
A schematic diagram of aging in the kidney. The aging process reflects alterations in all organs, including the kidney. The kidney compartment underwent different modifications at the molecular level, becoming senescent and increasingly immunogenic if grafted. Created with BioRender.com

From a pathophysiological viewpoint, these conditions reflect clinical and metabolic processes related to organ and immunological aging (Fig. 2).

Aging is defined as the decline of physiological integrity due to an accumulation of damage and deterioration of proteins and organelle functions [90]; its cellular counterpart, as described by Hayflick and Moorhead, is senescence [91].

Senescence is determined by a permanent decline in cell proliferation due to different stimuli (i.e., the accumulation of DNA damage, telomere shortening, high levels of reactive oxygen species, genetic mutations, chromatin remodeling, and mitochondrial dysfunction). Senescent cells also acquire a proinflammatory profile with the secretion of cytokines/chemokines, such as interleukin-6 (IL-6), matrix metalloproteinases, and growth factors [92].

Autophagy is also profoundly involved in kidney aging: this process, which is strictly mTOR-dependent, determines adequate protein and organelle degradation but declines with age, causing age-related waste accumulation in cells [93]; this accumulation results in increasing numbers of misfolded proteins and the formation of inclusion bodies and deformed organelles, with crucial impact on terminal cells (i.e., podocytes) [94]. In this context, both genetic and drug-induced mTOR and AMPK-ULK1 pathways may represent potential targets to increase autophagy and reduce organ aging [95].

Modification of protein folding also depends on heat shock proteins, a subgroup of chaperones. Barna et al. demonstrated that with aging, the master regulator of heat shock protein transcription (HSF1) decreases the ability to bind to heat shock protein genes upon stress [96]. Additionally, low-grade constitutive heat shock protein expression differs between standard and pathological allografts, suggesting a possible connection between aging, transplant outcome, and heat shock protein activity [97].

From a genetic point of view, some authors have investigated the role of age-related modifications in the kidney compartment. Rodwell et al. identified a pool of kidney-specific signatures that change expression in the cortex and the medulla with age; forty-nine age-regulated genes encode protein components of the extracellular matrix, all but four of which increase expression in old age. Considering the crucial role of the extracellular matrix in the filtration process via the basement membrane and its well-known decline with age, this study highlights the potential role of these age-related modifications in causing nonspecific injuries that may induce a proinflammatory niche that, in turn, activates innate and adaptive immune responses [98].

In this context, Franceschi et al. encompass organ and organism aging in “inflammaging,” defining this condition as the chronic, low-grade inflammation that occurs during aging and contributes to the pathogenesis of age-related diseases. According to this definition, cellular senescence, mitochondrial dysfunction, defective autophagy and mitophagy, and activation of the inflammasome are linked, with the additional contribution of metabolic inflammation driven by nutrient excess or overnutrition (the so-called “metaflammation”) [99]. The use of specific biomarkers (i.e., DNA methylation, glycomics, and metabolomics) may open the door to individual evaluation of the metabolic and inflammatory profile of donors and recipients, rewriting the parameters of “old” and “age” for grafts and patients, respectively.

Some examples of specific approaches are already available: Kimmel et al. applied single-cell sequencing to identify the upregulation of inflammatory pathways in old vs. young mice [100]; Elyahu et al. documented, through single-cell RNA sequencing and multidimensional protein analyses, a modification of the CD4 T-cell profile in aged mice, with alterations of regulatory, exhausted, and cytotoxic patterns and different expression of inflammatory cytokines (IL-27, IFNb, IL-6) [101].

All these data pave the way for a future evaluation of specific age-related organ modifications, allowing us to accurately characterize the detailed footprint of “aged” organs according to the objective biological age rather than the chronological one.

At the same time, these new molecular insights pose new questions, for example, in the case of donors with unfavorable biological age (based on the molecular analyses themselves) but with still adequately preserved renal function.

As mentioned above, we strongly recommend adopting a pragmatic approach, encompassing functional, histological, perfusion, and, in the near future, molecular information, to implement the decision-making process without constituting a barrier to organ acceptance, possibly considering dual kidney transplantation in high-risk settings (e.g., donor > 80 years old) to minimize the discard.

## Conclusions

Based on the confirmed results of kidney transplantation in improving the quality of life and survival of patients with end-stage kidney disease, the use of extended criteria donor organs appears to be a crucial issue in the transplantation field for expanding the donor pool. In the (not-so-distant) future, extended criteria donors may be treated with the previously mentioned techniques to obtain “young” and low tolerogenic tissues. To date, a combination of available therapeutic strategies and allocation policies, with a tailored evaluation for each patient on the waiting list according to their specific clinical characteristics, may improve results and expand and optimize extended criteria donor utilization.

## Data Availability

All data and datasets used and/or analyzed during the current study are available from the corresponding author on reasonable request.
